# Comparison of a Significant Decline in the Glomerular Filtration Rate between Ileal Conduit and Ileal Neobladder Urinary Diversions after Radical Cystectomy: A Propensity Score-Matched Analysis

**DOI:** 10.3390/jcm9072236

**Published:** 2020-07-14

**Authors:** Jihion Yu, Bumsik Hong, Jun-Young Park, Yongsoo Lee, Jai-Hyun Hwang, Yu-Gyeong Kong, Young-Kug Kim

**Affiliations:** 1 Department of Anesthesiology and Pain Medicine, Asan Medical Center, University of Ulsan College of Medicine, Seoul 05505, Korea; yujihion@gmail.com (J.Y.); parkjy@amc.seoul.kr (J.-Y.P.); thisisrio@naver.com (Y.L.); jhhwang11@hotmail.com (J.-H.H.); 2 Department of Urology, Asan Medical Center, University of Ulsan College of Medicine, Seoul 05505, Korea; bshong@amc.seoul.kr; 3 Department of Anesthesiology and Pain Medicine, Hangang Sacred Heart Hospital, Hallym University College of Medicine, Seoul 07247, Korea

**Keywords:** radical cystectomy, ileal conduit urinary diversion, ileal neobladder urinary diversion, glomerular filtration rate

## Abstract

Urinary diversion after radical cystectomy is associated with a risk of renal function impairment. A significant decline in the glomerular filtration rate (GFR) (i.e., a ≥30% decline in baseline GFR after 12 months) is associated with long-term renal function impairment. We compared the significant GFR decline between ileal conduit and ileal neobladder urinary diversions 12 months after radical cystectomy. We retrospectively included radical cystectomy patients. Propensity score-matched analysis was performed. The primary outcome was the incidence of a significant GFR decline in ileal conduit urinary diversion (ileal conduit group) and ileal neobladder urinary diversion (ileal neobladder group) 12 months after radical cystectomy. The secondary outcomes were the change of GFR and the incidence of end-stage renal disease (ESRD) in the two groups. After propensity score matching, the ileal conduit and neobladder groups had 117 patients each. The incidence of a significant GFR decline was not significantly different between ileal conduit and ileal neobladder groups (12.0% vs. 13.7%, *p* = 0.845). The change of GFR and ESRD incidence were not significantly different between the two groups (−8.4% vs. −9.7%, *p* = 0.480; 4.3% vs. 5.1%, *p* > 0.999, respectively). These results can provide important information on appropriate selection of the urinary diversion type in radical cystectomy.

## 1. Introduction

Radical cystectomy is widely used as the most effective treatment for muscle-invasive bladder cancer [1]. The two most frequently used methods for urinary diversion in radical cystectomy are ileal conduit urinary diversion and ileal neobladder urinary diversion. However, there is an ongoing debate regarding the selection of the appropriate urinary diversion type in radical cystectomy [2]. The ileal conduit urinary diversion has the advantages of having a less technically complicated procedure and shorter operative duration [3], while this has some disadvantages such as stomal stenosis, parastomal hernia, urinary incontinence, and negative body image due to external ostomy [2]. On the contrary, the ileal neobladder urinary diversion maintains anatomical voiding and can increase the quality of life of patients [4]. However, complications such as ureteroileal stricture, bladder outlet obstruction, and hydronephrosis can occur after ileal neobladder urinary diversion [5,6,7]. Therefore, the type of urinary diversion in radical cystectomy should be carefully selected considering patient characteristics and postoperative complications.

The impairment of renal function is an important risk factor for postoperative long-term morbidity and mortality [8]. Especially, a ≥30% or ≥40% decline in baseline glomerular filtration rate (GFR) after 12 months is a well-accepted surrogate for renal disease progression [9]. Additionally, a ≥30% or ≥40% decline in baseline GFR after 1 or 2 years is known to have a significant association with long-term renal function impairment such as end-stage renal disease (ESRD) and increases the risk of mortality [10]. Furthermore, both ileal conduit and ileal neobladder urinary diversions after radical cystectomy have a risk of chronic kidney disease [5,11,12]. Therefore, we consider that a significant decline in the GFR (i.e., a ≥30% decline in baseline GFR after 12 months) [9,10] can provide useful information on long-term renal function impairment after radical cystectomy. However, little is known regarding the comparison of a significant decline in the GFR between different urinary diversion types in radical cystectomy.

In this study, we compared the significant decline in the GFR between ileal conduit urinary diversion (ileal conduit group) and ileal neobladder urinary diversion (ileal neobladder group) 12 months after radical cystectomy. In addition, we compared the change of GFR and the incidence of ESRD between the two groups.

## 2. Materials and Methods

### 2.1. Patients

We retrospectively recruited the patients who underwent radical cystectomy with urinary diversion at the Asan Medical Center, Seoul, Republic of Korea between January 2007 and December 2018. We excluded patients who had incomplete medical records, were diagnosed as having ESRD, and underwent combined surgery with radical cystectomy. We reviewed the medical records and noted a significant decline in the GFR. The present study was approved by the institutional review board of the Asan Medical Center (approval no. 2020-0577). The requirement for written informed consent was waived by the institutional review board of the Asan Medical Center owing to the retrospective study design. All methods were carried out in accordance with relevant guidelines and regulations.

### 2.2. Intraoperative Protocols

General anesthesia was performed according to the standard technique used at the institution [13,14]. In brief, general anesthesia was induced with 1.5–2 mg/kg of propofol or 4–5 mg/kg of thiopental sodium with 0.5–0.8 mg/kg of rocuronium and maintained with 1.5–2.5 vol% sevoflurane and 50% oxygen. Electrocardiography, peripheral oxygen saturation, end-tidal carbon dioxide concentration, continuous arterial blood pressure, central venous pressure, bispectral index, and body temperature were monitored in all patients. End-tidal carbon dioxide concentration was maintained at 35–40 cm H_2_O by maintaining the tidal volume in the range of 8–10 mL per ideal body weight and the respiratory rate in the range of 10–14 cycles/min. Fluid and vasopressors or inotropes were administered to maintain the mean arterial blood pressure above 65 mmHg. The fluids administered were crystalloids such as plasma solution A (CJ Pharmaceutical, Seoul, Korea) or lactated Ringer’s solution and colloids such as 5% albumin or 6% hydroxyethyl starch. The administered vasopressors or inotropes were ephedrine, phenylephrine, or norepinephrine. When hemoglobin concentration was less than 8 g/dL, red blood cell transfusion was performed. The neostigmine–glycopyrrolate mixture or sugammadex was used as a neuromuscular reversal agent. Patient-controlled analgesia via the intravenous route was used to manage postoperative pain.

Radical cystectomy and pelvic lymphadenectomy were performed according to the standard technique used at the institution. Details of the surgical method have been previously described [15,16]. The choice of either standard or extended pelvic lymphadenectomy was made by the surgeons. Standard pelvic lymphadenectomy included the perivesical, obturator, hypogastric, external iliac, and distal common iliac lymph nodes. Extended lymphadenectomy included the lymph nodes at the level of the proximal common iliac artery, distal aorta, and inferior vena cava. Subsequently, ileal conduit urinary diversion or ileal neobladder urinary diversion was performed according to the decision of the surgeons. The ileal conduit urinary diversion was performed as follows: the ileal conduit was made by harvesting 20 cm of the ileal segment. A total of 15 cm of the distal ileum was preserved and re-anastomosed. Both ureters were transplanted into the ileal conduit. The stoma was formed on the chosen site of abdomen surface. The ileal neobladder urinary diversion was performed as follows: a specific length of the distal ileum (15–20 cm away from the ileocecal valve) according to the type of ileal neobladder was harvested and an orthotopic continent diversion was created. The remnant small bowel was re-continued by ileo-ileostomy.

### 2.3. Definition of a Significant Decline in the GFR

A significant decline in the GFR was defined as a decline of ≥30% from the baseline GFR value, which was estimated within 7 days before the surgery [9,10], and evaluated 12 months after radical cystectomy with ileal conduit urinary diversion or ileal neobladder urinary diversion.

### 2.4. Primary and Secondary Outcomes

The primary outcome was the incidence of a significant GFR decline in ileal conduit group and ileal neobladder group 12 months after radical cystectomy. The GFR was evaluated using the Chronic Kidney Disease Epidemiology Collaboration (CKD-EPI) equation: GFR_CKD-EPI_ = 141 × (minimum of standardized serum creatinine [mg/dL]/κ or 1)^α^ × (maximum of standardized serum creatinine [mg/dL]/κ or 1)^−1.209^ × 0.993 ^age^ × (1.018 if female), where κ is 0.7 for women and 0.9 for men and α is −0.329 for women and −0.411 for men [17,18].

The secondary outcomes were the change of GFR and the incidence of ESRD in the two groups. The change of GFR was defined as (GFR at 12 months after radical cystectomy—GFR at baseline)/GFR at baseline × 100. The ESRD, which was assessed from 12 months after radical cystectomy to the last follow-up, was defined by the initiation of dialysis or a GFR < 15 mL/min/1.73 m^2^ [19,20].

### 2.5. Data Collection and Definitions

Patient demographics and clinical characteristics included sex, age, body mass index, American Society of Anesthesiologist Physical Status, comorbidities (diabetes mellitus, hypertension, atrial fibrillation, coronary artery disease, cerebrovascular disease, chronic obstructive pulmonary disease, and chronic kidney disease), preoperative medications (angiotensin-converting enzyme inhibitor or angiotensin II receptor blocker, diuretic, calcium channel blocker, beta-blocker, plavix, and aspirin), tumor stage, tumor grade, neo-adjuvant chemotherapy application, hydronephrosis, and preoperative laboratory data (white blood cell count, neutrophil percentage, lymphocyte percentage, hemoglobin concentration, platelet count, serum creatinine level, GFR, serum albumin level, aspartate aminotransferase, alanine aminotransferase, serum sodium level, serum potassium level, serum chloride level, and serum uric acid level). Intraoperative data included the operation time, infused crystalloid and colloid amounts, and red blood cell transfusion rate. Postoperative outcomes included hospital stay (the number of days from surgery to discharge), intensive care unit admission rate, intensive care unit stay, the incidence of acute kidney injury, the incidence of postoperative ureterointestinal stricture, the rate of adjuvant chemotherapy, the incidence of a significant decline in the GFR, the change of GFR, and the incidence of ESRD.

Coronary artery disease was defined as a history of angina, myocardial infarction, interventional angioplasty, or coronary artery bypass graft surgery. Cerebrovascular disease was defined as a history of cerebrovascular accident, stroke, or transient ischemic accident. Chronic kidney disease was defined as a GFR of <60 mL/min/1.73 m^2^. Tumor stage and tumor grade were confirmed by genitourinary pathologists according to the 2010 American Joint Committee on Cancer tumor-node-metastasis staging system [21] and the 2016 World Health Organization grading system [22]. Neo-adjuvant chemotherapy was performed according to the decision of surgeons and oncologists. The neo-adjuvant chemotherapy regimen used was a combination of cisplatin and gemcitabine; methotrexate and vinblastine; cisplatin, doxorubicin, and sulfate; or carboplatin and gemcitabine. Postoperative acute kidney injury was defined by the Kidney Disease: Improving Global Outcomes criteria: the serum creatinine level increases by 0.3 mg/dL from the baseline value within 2 postoperative days or 50% from the baseline value within 7 postoperative days [23,24].

### 2.6. Statistical Analysis

Continuous variables were analyzed using either Student’s t-test or Mann–Whitney U-test and reported as mean ± standard deviation. Categorical variables were analyzed using chi-square test or Fisher’s exact test and reported as counts and percentages. Before propensity score matching, all variables including patient characteristics, tumor-related data, and preoperative laboratory data were compared between ileal conduit group and ileal neobladder group. The 1:1 propensity score-matched analysis was performed by the nearest neighbor method with a 0.2-caliper size to ascertain the impact of urinary diversion type on a significant decline in the GFR. The propensity score was determined by multiple logistic regression analysis using the following variables: sex, age, body mass index, American Society of Anesthesiologist Physical Status, comorbidities (diabetes mellitus, hypertension, atrial fibrillation, coronary artery disease, cerebrovascular disease, chronic obstructive pulmonary disease, and chronic kidney disease), preoperative medications (angiotensin-converting enzyme inhibitor or angiotensin II receptor blocker, diuretic, calcium channel blocker, beta-blocker, plavix, and aspirin), tumor stage, tumor grade, neo-adjuvant chemotherapy application, hydronephrosis, white blood cell count, neutrophil percentage, lymphocyte percentage, hemoglobin concentration, platelet count, serum creatinine level, GFR, serum albumin level, aspartate aminotransferase, alanine aminotransferase, serum sodium level, serum potassium level, serum chloride level, and serum uric acid level. The standardized mean difference was calculated to determine the balance between the two groups and the balance between the two groups was considered sufficient when the standardized mean difference was less than 0.2. After a 1:1 propensity score matching, we compared the intraoperative data and postoperative outcomes using paired t-test for continuous variables and McNemar’s test for categorical variables. Values of *p* < 0.05 were considered statistically significant. All statistical analyses were performed using SPSS^®^ software version 21.0 (IBM, Armonk, NY, USA).

## 3. Results

**Figure 1:**
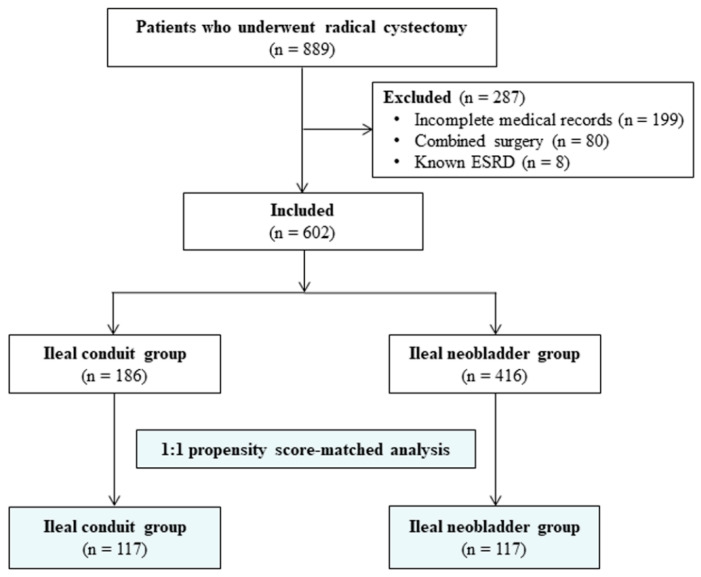
Flowchart of the study’s patients: 889 patients who underwent radical cystectomy were evaluated, and 602 patients were included in the study. Patients were dichotomized according to urinary diversion type, and a propensity score-matched analysis was performed.

Of 889 patients who underwent radical cystectomy between January 2007 and December 2018, 602 patients were included. Two hundred and eighty-seven patients were excluded (199 patients owing to incomplete medical records, 80 owing to combined surgery with radical cystectomy, and 8 owing to known ESRD). Of the 602 patients included, 186 patients underwent ileal conduit urinary diversion and 416 patients underwent ileal neobladder urinary diversion after radical cystectomy. After 1:1 propensity score matching, patients were divided into ileal conduit group (*n* = 117) and ileal neobladder group (*n* = 117) (Figure 1).

Before 1:1 propensity score matching, there were no significant differences in preoperative serum creatinine level and GFR before and after neo-adjuvant chemotherapy (1.00 ± 0.30 mg/dL vs. 1.06 ± 0.34 mg/dL, *p* = 0.211; 92.8 ± 16.6 mL/min/1.73 m^2^ vs. 89.2 ± 18.0 mL/min/1.73 m^2^, *p* = 0.109, respectively).

Before 1:1 propensity score matching, sex, age, body mass index, American Society of Anesthesiologist Physical Status, hypertension, atrial fibrillation, cerebrovascular disease, chronic kidney disease, diuretic, calcium channel blocker, plavix, aspirin, hydronephrosis, hemoglobin concentration, serum creatinine level, GFR, serum albumin level, aspartate aminotransferase, alanine aminotransferase, serum chloride level, and serum uric acid level were significantly different between the two groups (Table 1). After 1:1 propensity score matching, all covariates were not significantly different and were well-balanced with a standardized mean difference <0.2 between the two groups (Table 1).

After 1:1 propensity score matching, the intraoperative data including operation time, crystalloid and colloid amounts, and red blood cell transfusion rate were not significantly different between the two groups (Table 2). In addition, after propensity score matching, postoperative outcomes including the hospital stay, intensive care unit admission rate, intensive care unit stay, incidence of acute kidney injury, and adjuvant chemotherapy were not significantly different between the two groups (Table 2). The incidence of ureterointestinal stricture was significantly higher in the ileal neobladder group than in the ileal conduit group (*p* = 0.031) (Table 2).

**Figure 2:**
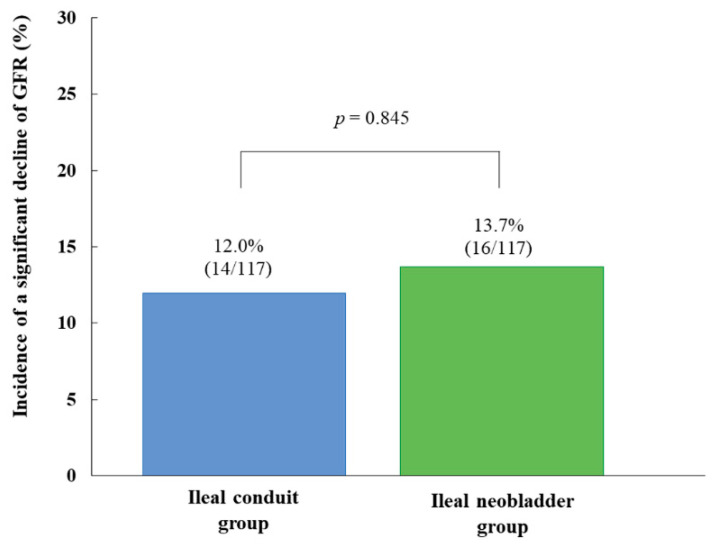
Comparison of the incidence of a significant decline in the GFR between ileal conduit group and ileal neobladder group 12 months after radical cystectomy. A significant decline in the GFR was defined as a ≥30% decline in baseline GFR after 12 months. Note that the incidence of a significant decline in the GFR was not significantly different between the two groups. GFR, glomerular filtration rate.

The overall incidence of a significant decline in the GFR 12 months after radical cystectomy was 12.8% (30/234), and there was no significant difference in the incidence of a significant decline in the GFR between ileal conduit group and ileal neobladder group (12.0% (14/117) vs. 13.7% (16/117), *p* = 0.845) (Figure 2).

**Figure 3:**
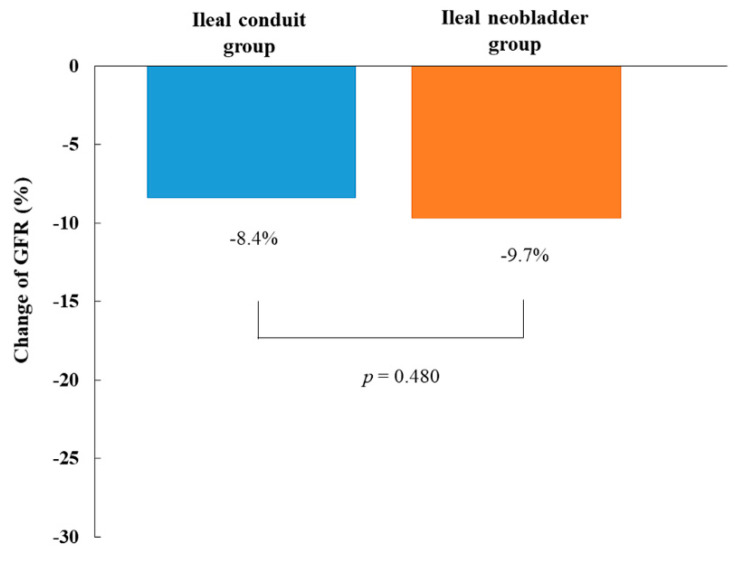
Comparison of the change of GFR between ileal conduit group and ileal neobladder group 12 months after radical cystectomy. The change of GFR was defined as (GFR at 12 months after radical cystectomy—GFR at baseline)/GFR at baseline × 100. Note that the change of GFR was not significantly different between the two groups. GFR, glomerular filtration rate.

The change of GFR showed no significant difference between ileal conduit group and ileal neobladder group (−8.4 ± −16.2% vs. −9.7 ± −13.3%, *p* = 0.480) (Figure 3).

**Figure 4:**
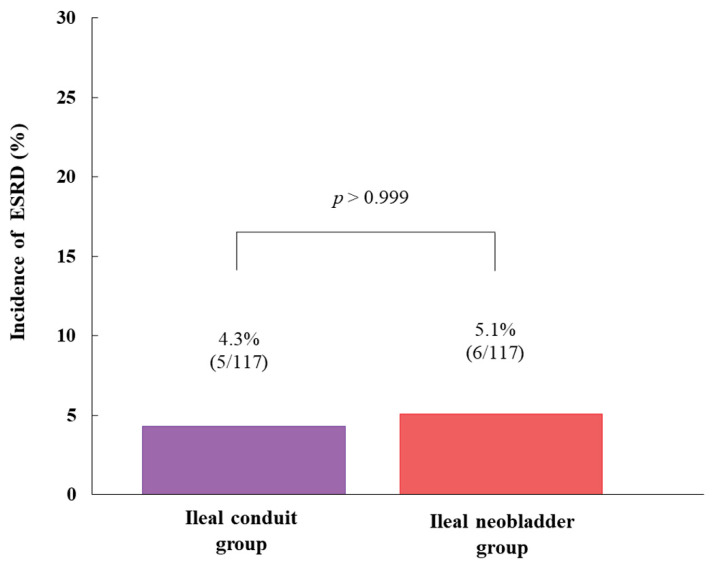
Comparison of the incidence of ESRD between ileal conduit group and ileal neobladder group 12 months after radical cystectomy. Note that the incidence of ESRD was not significantly different between the two groups. ESRD, end-stage renal disease.

The incidence of ESRD was not significantly different between the ileal conduit group and the ileal neobladder group (4.3% (5/117) vs. 5.1% (6/117), *p* > 0.999) (Figure 4). The mean duration of ESRD follow-up assessment after radical cystectomy was 80.8 ± 42.0 months. The incidence of ESRD was significantly higher in patients with a significant decline in the GFR than in those without a significant decline in the GFR (20.0% (6/30) vs. 2.5% (5/204), *p* = 0.001).

The one-year postoperative data such as hemoglobin, sodium, potassium, chloride, uric acid, creatinine, GFR, and mortality were not significantly different between the ileal conduit group and the ileal neobladder group (Table 3).

## 4. Discussion

The present study showed that 12.8% of the patients developed a significant decline in the GFR 12 months after radical cystectomy with urinary diversion. However, the incidence of a significant decline in the GFR was not significantly different between ileal conduit urinary diversion and ileal neobladder urinary diversion. In addition, the change of GFR and the incidence of ESRD were not significantly different between the two groups. The ESRD incidence was significantly higher in patients with a significant GFR decline than in those without a significant GFR decline. To the best of our knowledge, this is the first study to demonstrate that the urinary diversion type is not associated with a significant decline in the GFR after radical cystectomy, which can lead to the progression of postoperative ESRD.

Radical cystectomy is the most effective treatment for muscle-invasive bladder cancer. After the bladder is removed, the reconstruction of the lower urinary tract is necessary for drainage of urine. Therefore, urinary diversion is an important surgical procedure to replace the bladder. The ileal conduit urinary diversion creates the conduit using the ileum, and then makes a stoma in the abdominal wall. On the contrary, the ileal neobladder urinary diversion creates a new bladder typically using the ileum [2,25]. Since Bricker reported the use of the ileum as a urinary conduit in 1950, the ileal conduit urinary diversion was considered as a standard treatment of urinary diversion until recently [26]. A commonly considered advantage of ileal conduit urinary diversion is the shorter operative duration, which is closely associated with lower postoperative morbidity and mortality in patients with old age and multiple comorbidities [27,28]. However, the ileal conduit urinary diversion has several disadvantages including an external stoma, stomal stenosis, and para-stomal hernia [2]. Meanwhile, the ileal neobladder urinary diversion enables urine storage and urination similar to that observed with the normal bladder. The quality of life of the patients who underwent ileal neobladder urinary diversion could be better improved than that of the patients who underwent ileal conduit urinary diversion [4]. Based on these considerations, the ileal neobladder urinary diversion is considered as a more ideal form of urinary diversion [29]. However, there is still a debate as to which is the appropriate type of urinary diversion to choose in radical cystectomy, because there are various factors to consider, including surgeon preferences, patient characteristics, bowel availability, renal function, disease progression, and economic status [2]. Therefore, the type of urinary diversion in radical cystectomy needs to be cautiously selected, taking into account various factors.

Renal dysfunction can occur after both types of urinary diversions in patients undergoing radical cystectomy. However, most previous studies did not compare the change in renal function between the two types of urinary diversions and evaluated mainly only early renal outcomes such as acute kidney injury [11,30,31,32]. Therefore, little is known about the comparison of renal function at 12 months postoperatively between ileal conduit urinary diversion and ileal neobladder urinary diversion. In this study, we compared the significant GFR decline and the GFR change between ileal conduit and ileal neobladder urinary diversions 12 months after radical cystectomy. We found no significant differences in the significant GFR decline and the GFR change between the two types of urinary diversion 12 months after radical cystectomy. Inconsistent with our results, in a previous study, serum creatinine levels between postoperative 1 and 2 years were lower in patients who underwent ileal conduit urinary diversion than in those who underwent ileal neobladder urinary diversion [33]. Since the ileal conduit urinary diversion is done by using a small segment of the ileum, the urine remains during very short periods of time in the ileal conduit [34,35]. Subsequently, the reabsorption of urine creatinine and urea in the ileal conduit may be relatively low. Therefore, the ileal conduit urinary diversion is considered to be more tolerable to postoperative renal dysfunction than the ileal neobladder urinary diversion [34,35]. On the contrary, in another previous study that assessed the renal function 10 years postoperatively in patients who underwent urinary diversion, the impairment in renal function such as a decrease in GFR > 10 mL/min/1.73 m^2^ occurred more in ileal conduit urinary diversion than in ileal neobladder urinary diversion [5]. This may be associated with higher incidences of urinary tract obstruction and comorbidities such as diabetes mellitus and hypertension in patients who underwent ileal conduit urinary diversion [5,36]. Taken together, these inconsistent findings about postoperative renal function between the two types of urinary diversions might, at least in part, be due to uncontrolled patient characteristics and intraoperative variables between ileal conduit and ileal neobladder urinary diversions. Using the propensity score matching analysis, our study compared only controlled patients without significant differences in patient characteristics such as comorbidities and intraoperative variables such as operation time and red blood cell transfusion rate between the two types of urinary diversions. Based on these considerations, our results seem to be more reliable than other results in previous studies. Therefore, we concluded that the type of urinary diversion may have little effect on postoperative renal complications in radical cystectomy.

A significant decline in the GFR (i.e., a ≥30% decline in baseline GFR after 12 months) has been regarded as a useful indicator of renal dysfunction for a relatively shorter period, instead of ESRD and doubling of serum creatinine levels (i.e., an approximately 57% decline in baseline GFR), as an end point of chronic kidney disease progression [9,10]. Through early detection of a significant decline in the GFR, early intervention for renal dysfunction is possible, and an improvement of the treatment effect estimation could be achieved. In this study, the significant decline in the GFR after 12 months showed a significant association with ESRD after radical cystectomy with urinary diversion. These results suggest that the significant decline in the GFR after 12 months can provide important information for evaluating long-term renal outcomes such as ESRD in radical cystectomy.

We found that the incidence of ESRD was not significantly different between the two types of urinary diversion. Similar to our study, the study by Zabell et al. retrospectively compared the incidence of ESRD between ileal conduit and ileal neobladder urinary diversions and demonstrated that the urinary diversion type did not affect the incidence of ESRD after radical cystectomy [37]. Importantly, in our study, patient characteristics and intraoperative variables were strictly controlled using the propensity score matching analysis and then resulted in no significant difference in ESRD incidence. Therefore, we concluded that the urinary diversion type may not influence long-term renal outcomes such as ESRD as well as a significant decline in the GFR in radical cystectomy.

This study had several limitations. First, its retrospective design was a limitation. The possibility of other confounding factors that could influence the renal outcomes cannot be excluded. However, we considered almost all possible confounding factors such as comorbidities and preoperative laboratory data, which may have affected a significant decline in the GFR in radical cystectomy. Furthermore, we performed a propensity score matching analysis for major confounding factors. Therefore, we consider that the selection bias may be minimized in the present study. Second, because our study was performed by highly experienced surgeons at a large single center, the results need to be interpreted carefully.

## 5. Conclusions

The incidence of a significant decline in the GFR was not significantly different between ileal conduit urinary diversion and ileal neobladder urinary diversion 12 months after radical cystectomy. Moreover, the change of GFR and the incidence of ESRD were not significantly different between the two types of urinary diversion after radical cystectomy. These results can provide important information on appropriate selection of the urinary diversion type in radical cystectomy.

## Figures and Tables

**Table 1:** Preoperative variables before and after propensity score matching.

	Before Propensity Score Matching				After Propensity Score Matching			
Variables	Ileal Conduit Group (*n* = 186)	Ileal Neobladder Group (*n* = 416)	SMD	*p*-Value	Ileal Conduit Group (*n* = 117)	Ileal Neobladder Group (*n* = 117)	SMD	*p*-Value
Sex (male)	125 (67.2)	387 (93.0)	1.013	<0.001	21 (17.9)	22 (18.8)	0.034	>0.999
Age (years)	69 ± 10	61 ± 9	−0.812	<0.001	67 ± 10	66 ± 9	−0.138	0.212
Body mass index (kg/m^2^)	23.7 ± 3.1	24.6 ± 3.1	0.277	0.002	23.8 ± 3.1	23.8 ± 3.3	−0.011	0.931
ASA Physical Status			−0.335	0.002			<0.001	>0.999
≤2	160 (86.0)	391 (94.0)			105 (89.7)	105 (89.7)		
3	26 (14.0)	25 (6.0)			12 (10.3)	12 (10.3)		
Diabetes mellitus	37 (19.9)	70 (16.8)	−0.082	0.419	20 (17.1)	20 (17.1)	<0.001	>0.999
Hypertension	96 (51.6)	153 (36.5)	−0.307	<0.001	52 (44.4)	45 (38.5)	−0.124	0.401
Atrial fibrillation	8 (4.3)	5 (1.2)	−0.284	0.028	2 (1.7)	1 (0.9)	−0.078	>0.999
Coronary artery disease	12 (6.5)	14 (3.4)	−0.171	0.126	6 (5.1)	6 (5.1)	<0.001	>0.999
Cerebrovascular disease	9 (4.8)	7 (1.7)	−0.245	0.032	4 (3.4)	5 (4.3)	0.066	>0.999
COPD	5 (2.7)	15 (3.6)	0.049	0.633	5 (4.3)	2 (1.7)	−0.137	0.453
Chronic kidney disease	17 (9.1)	7 (1.7)	−0.499	<0.001	3 (2.6)	6 (5.1)	0.139	0.508
Medications								
ACEi or ARB	33 (17.7)	51 (12.3)	−0.167	0.076	17 (14.5)	13 (11.1)	−0.104	0.503
Diuretic	14 (7.5)	11 (2.6)	−0.304	0.008	5 (4.3)	5 (4.3)	<0.001	>0.999
Calcium channel blocker	62 (33.3)	98 (23.6)	−0.230	0.013	32 (27.4)	30 (25.6)	−0.040	0.878
Beta blocker	14 (7.5)	20 (4.8)	−0.127	0.251	8 (6.8)	7 (6.0)	−0.040	>0.999
Plavix	8 (4.3)	3 (0.7)	−0.423	0.005	2 (1.7)	2 (1.7)	<0.001	>0.999
Aspirin	22 (11.8)	18 (4.3)	−0.368	0.001	9 (7.7)	7 (6.0)	−0.084	0.774
Tumor stage			−0.118	0.198			<0.001	>0.999
≤2	129 (69.4)	310 (74.5)			85 (72.6)	85 (72.6)		
≥3	57 (30.6)	106 (25.5)			32 (27.4)	32 (27.4)		
Tumor grade			−0.013	>0.999			<0.001	>0.999
2	8 (4.3)	19 (4.6)			5 (4.3)	5 (4.3)		
3	178 (95.7)	397 (95.4)			112 (95.7)	112 (95.7)		
Neo-adjuvant chemotherapy	39 (21.0)	79 (19.0)	−0.050	0.580	24 (20.5)	28 (23.9)	0.087	0.618
Hydronephrosis	36 (19.4)	28 (6.7)	−0.503	<0.001	17 (14.5)	13 (11.1)	−0.136	0.572
Preoperative laboratory data								
White blood cell (10^3^/µL)	6.5 ± 2.2	6.7 ± 2.5	0.046	0.589	6.6 ± 2.3	6.6 ± 3.0	0.026	0.846
Neutrophil (%)	57.9 ± 11.8	56.5 ± 11.5	−0.124	0.166	57.1 ± 11.2	56.9 ± 11.2	−0.019	0.887
Lymphocyte (%)	29.9 ± 10.3	31.3 ± 9.9	0.139	0.119	30.4 ± 9.5	30.4 ± 10.3	0.003	0.980
Hemoglobin (g/dL)	11.7 ± 1.8	12.8 ± 1.9	0.608	<0.001	12.0 ± 1.8	12.1 ± 1.8	0.050	0.663
Platelet (10^3^/µL)	237.0 ± 83.9	245.4 ± 80.7	0.104	0.242	236.1 ± 84.9	248.6 ± 90.9	0.155	0.284
Creatinine (mg/dL)	1.1 ± 0.4	1.0 ± 0.2	−0.503	<0.001	1.0 ± 0.3	1.0 ± 0.3	−0.018	0.921
GFR (mL/min/1.73 m^2^)	86.8 ± 17.7	97.4 ± 17.1	0.619	<0.001	91.0 ± 18.3	91.5 ± 19.0	0.032	0.820
Albumin (g/dL)	3.6 ± 0.4	3.9 ± 0.4	0.721	<0.001	3.7 ± 0.4	3.6 ± 0.4	−0.066	0.612
AST (IU/L)	21.3 ± 7.7	22.9 ± 8.7	0.178	0.038	21.6 ± 8.0	20.9 ± 6.5	−0.082	0.431
ALT (IU/L)	17.2 ± 9.8	22.5 ± 15.7	0.337	<0.001	18.3 ± 9.9	17.6 ± 11.8	−0.042	0.636
Sodium (mmol/L)	140 ± 3	140 ± 3	0.016	0.859	140 ± 3	140 ± 2	−0.038	0.762
Potassium (mmol/L)	4.3 ± 0.4	4.4 ± 0.4	0.043	0.638	4.3 ± 0.3	4.3 ± 0.4	−0.029	0.823
Chloride (mmol/L)	105 ± 4	104 ± 3	−0.187	0.042	105 ± 3	105 ± 3	−0.081	0.513
Uric acid (mg/dL)	5.1 ± 1.6	5.4 ± 1.5	0.194	0.032	5.2 ± 1.4	5.2 ± 1.6	0.060	0.639

Continuous variables are presented as mean ± standard deviation and categorical variables as number (percentage). SMD, standardized mean difference; ASA, American Society of Anesthesiologists; COPD, chronic obstructive pulmonary disease; ACEi, angiotensin-converting enzyme inhibitor; ARB, angiotensin II receptor blocker; GFR, glomerular filtration rate; AST, aspartate aminotransferase; ALT, alanine aminotransferase.

**Table 2:** Intraoperative data and postoperative outcomes after propensity score matching.

Variables	All Patients (*n* = 234)	Ileal Conduit Group (*n* = 117)	Ileal Neobladder Group (*n* = 117)	*p*-Value
Operation time (minute)	435 ± 111	434 ± 118	436 ± 104	0.929
Crystalloid amount (mL)	3542 ± 1396	3637 ± 1629	3446 ± 1114	0.314
Colloid amount (mL)	547 ± 432	579 ± 513	515 ± 403	0.285
Red blood cell transfusion rate	128 (54.7)	65 (55.6)	63 (53.8)	0.896
Hospital stay (days)	26 ± 28	25 ± 33	27 ± 21	0.628
Intensive care unit admission rate	54 (23.1)	30 (25.6)	24 (20.5)	0.392
Intensive care unit stay (days)	0.2 ± 0.5	0.3 ± 0.5	0.2 ± 0.4	0.240
Acute kidney injury	64 (27.4)	35 (29.9)	29 (24.8)	0.451
Ureterointestinal stricture	20 (8.5)	5 (4.3)	15 (12.8)	0.031
Adjuvant chemotherapy	99 (42.3)	52 (44.4)	47 (40.2)	0.615

Variables are presented as mean ± standard deviation or number (percentage).

**Table 3:** One-year postoperative data after propensity score matching.

Variables	All Patients (*n* = 234)	Ileal Conduit Group (*n* = 117)	Ileal Neobladder Group (*n* = 117)	*p*-Value
Hemoglobin (g/dL)	12.5 ± 2.0	12.6 ± 2.0	12.3 ± 2.0	0.294
Sodium (mmol/L)	138 ± 3	138 ± 3	138 ± 4	0.866
Potassium (mmol/L)	4.4 ± 0.4	4.4 ± 0.4	4.4 ± 0.5	0.880
Chloride (mmol/L)	105 ± 4	104 ± 3	105 ± 4	0.475
Uric acid (mg/dL)	5.5 ± 1.4	5.6 ± 1.4	5.5 ± 1.5	0.701
Creatinine (mg/dL)	1.1 ± 0.4	1.1 ± 0.4	1.2 ± 0.4	0.289
GFR (mL/min/1.73 m^2^)	82.0 ± 17.1	82.6 ± 19.8	81.4 ± 13.8	0.586
Mortality	5 (2.1)	2 (1.7)	3 (2.6)	>0.999

Variables are presented as mean ± standard deviation or number (percentage). GFR, glomerular filtration rate.
